# Involvement of RFC3 in tamoxifen resistance in ER-positive breast cancer through the cell cycle

**DOI:** 10.18632/aging.205260

**Published:** 2023-12-06

**Authors:** Jintao Zhu, Lei Ye, Shishen Sun, Jie Yuan, Jianfeng Huang, Zhiqiang Zeng

**Affiliations:** 1 Department of Breast, Foshan Fosun Chancheng Hospital, Foshan, Guangdong, China; 2 Foshan Clinical Medical School of Guangzhou University of Chinese Medicine, Foshan, Guangdong, China; 3 Department of General Surgery, Foshan Fosun Chancheng Hospital, Foshan, Guangdong, China

**Keywords:** RFC3, breast cancer, ER-positive, tamoxifen resistance, prognosis, cell cycle

## Abstract

Since the establishment of the molecular subtyping system, ER positive breast cancer was considered to be the most prevalent type of breast cancer, and endocrine therapy was a very important solution. However, numerous studies have shown that the cell cycle plays a key role in the progression and metastasis of breast cancer. The present study showed that RFC3 was involved in the cell cycle through DNA replication. Furthermore, RFC3 expression was significantly higher in breast cancer-resistant cells than in parental cells, which correlated with the cell cycle. We confirmed these results by established drug-resistant cell lines for breast cancer, raw letter analysis and immunohistochemical analysis of primary and recurrent tissues from three ER+ breast cancers. In addition, analysis of the results through an online database revealed that RFC3 expression was significantly associated with poor prognosis in ER+ breast cancer. We also demonstrated that in ER positive breast cancer-resistant cells, knockdown of RFC3 blocked the S-phase of cells and significantly attenuated cell proliferation, migration and invasion. Furthermore, RFC3 overexpression in ER positive breast cancer cells enhanced cell proliferation, migration and invasion. Taking all these findings into account, we could conclude that RFC3 was involved in endocrine resistance in breast cancer through the cell cycle. Thus, RFC3 may be a target to address endocrine therapy resistance in ER positive breast cancer and may be an independent prognostic factor in ER positive breast cancer.

## INTRODUCTION

Breast cancer is the most common major cancer and the most prevalent cancer in women [1]. Several molecular subtypes of breast cancer have been classified [2]. The molecular subtype that accounts for 60–70% of the total incidence of breast cancer is the luminal type, which includes luminal A and luminal B and is the most common molecular subtype [3]. In luminal-type breast cancers, primary resistance is acquired or secondary resistance occurs in 70–80% of patients [4], because of the high proportion of ER+. More and more drug resistance mechanisms have been reported, including changes in ER-related signal transduction, epigenetic regulators, tumor microenvironment and nutritional metabolism [5, 6]. Therefore, it is essential to understand the underlying molecular mechanisms involved in endocrine resistance in ER+ breast cancer.

The cell cycle consists of the G1-phase (presynthesis), S-phase (DNA synthesis), G2-phase (late synthesis) and M phase (mitosis) [7]. Cell cycle regulation has important implications for tumor cell proliferation, metastasis and recurrence [8]. In current cancer therapy, cell cycle regulation is mainly concerned with controlling the expression of relevant genes and the activity of intracellular enzymes, proteins or signaling factors [9–11].

Tamoxifen (TAM) is a selective estrogen receptor modulator (SERM) for hormone-dependent breast cancer [12]. It competes with estrogen to form a stable complex that blocks the action of estrogen, thereby preventing the proliferation of breast cancer cells [13]. The clinical use of TAM has brought about significant prognostic improvements in patients with estrogen-positive breast cancer [14]. However, TAM resistance is a challenge for clinical treatment today. Tamoxifen resistance may be closely related to mutations in the ERα gene, expression levels of coregulatory factors and enhanced biological function [15, 16].

Replication Factor C (RFC), first purified from extracts of human cervical cancer HeLa cells, acts as an important factor involved in DNA replication [17, 18]. rFC3 is one of the five subunits of RFC (RFC1–5) and can act as a primer to recognize DNA polymerases, thus providing efficient detection and diagnostic functions [19, 20]. In recent years, RFC3 overexpression has been shown to have a critical impact on the proliferation, metastasis and prognosis of esophageal adenocarcinoma, hepatocellular carcinoma and ovarian cancer [21–23]. Although upregulation of RFC3 has been reported in several cancers, a correlation between RFC3 expression and progression of ER-positive breast cancer has not been reported. Therefore, the potential mechanism and role of RFC3 in ER-positive breast cancers should be described.

In the present study, we provide evidence that RFC3 expression is upregulated in resistant breast cancer cells and that RFC3 overexpression leads to poor prognosis in breast cancer patients. We also demonstrate that RFC3 overexpression promotes the proliferation, migration and invasion of breast cancer cells, while RFC3 knockdown attenuates the proliferation, migration and invasion of resistant breast cancer cells.

## MATERIALS AND METHODS

### Cellular and patient pathological tissue

The ER+ breast cancer cell line MCF-7 was used in this study, and the cells were purchased from Guangzhou Saiku Biotechnology Co. The pathological tissue samples used in this study were from patients with recurrent ER-positive breast cancer treated with endocrine therapy from March 2015 to September 2021 at the Foshan School of Clinical Medicine, Guangzhou University of Chinese Medicine, Foshan, China. Three matched pairs of pathological specimens of primary tumor and recurrent endocrine therapy were collected from the pathology department of the institution. Informed consent was obtained from the patients and approved by the Ethics Committee of Foshan Clinical School of Medicine, Guangzhou University of Chinese Medicine at the time of collection and application of pathological tissues.

### Culture of drug-resistant cells and organoids

MCF-7 cells were cultured in DMEM, induced by a low gradient of tamoxifen with the recommended supplements, in a 37°C 5% CO_2_ incubator at 37°C to obtain MCF-7R resistant strains. MCF-7 cells were combined with Matrigel and cultured in specific organoid medium, observed for daily growth and passaged to ultimately obtain MCF-7 organoids.

### RNA extraction, reverse transcription and quantitative real-time PCR

Total RNA was extracted from intracellular cells using TRIzol reagent and reverse transcribed using the Prime Script RT kit according to the manufacturer’s instructions. The reverse transcription PCR was programmed as follows: 95°C for 2 minutes, 40 cycles, 95°C for 15 seconds, 60°C for 30 seconds 60°C for 1 minute, and 95°C for 15 seconds. RT-PCR products were analyzed by 2.0% agarose gel electrophoresis. Real-time quantitative PCR was performed using SYBR PCR premix. The following primers were used: RFC3-F: 5′-TGATCCCACCTATTCGTAGT-3′; RFC3-R: 5′-CAGTCTCCCTCAGATACACC-3′; ACTIN-F: 5′-TGACGTGGACATCCGCAAAG-3′; ACTIN-R: 5′-CTGGAAGGTGGACAGCGAGG-3′.

### Western blotting

Cells were lysed in sample buffer, and protein concentrations were measured using a BCA kit. Equal amounts of protein were separated on 10% polyacrylamide SDS gels (SDS-PAGE), transferred to polyvinylidene difluoride (PVDF) membranes, and lysed with anti-RFC3 antibodies, ZO-1, E-calmodulin, N-calmodulin, and Vimentin, followed by peroxidase-coupled secondary antibodies. Signals were then visualized by enhanced chemiluminescence kits according to the manufacturer’s instructions. The anti-GAPDH antibody was used as a top sample control.

### Transcriptome sequencing and raw signal analysis

MCF-7-like organs were treated with tamoxifen and then subjected to RNA extraction and transcriptome sequencing analysis. A specific cDNA library was obtained by QPCR enrichment as described above. The library was sequenced by an Illumina HiSeq 4000 sequencer. The collated transcripts were compared with public databases such as Gene Ontology (GO) [24] and Kyoto Encyclopedia of Genes and Genomes (KEGG) [25] through the search tool (BLAST) BLASTn and BLASTx programs based on a local comparison algorithm. Expression profile data for wild-type and TAM-tolerant cells were made available through the GEO database (https://www.ncbi.nlm.nih.gov/geo/) [26]. Data were cleaned and normalized using R3.6.3 and the other support packages dplyr, and differential gene analysis was performed using the ggPlot2 package.

### CCK-8 experiment and ATP activity assay experiment

Cells were treated with PBS solution and then subjected to 0.25% trypsin breakdown to form a cell suspension. The cells were then cultured in DMEM containing tamoxifen at different concentrations and further incubated at 37°C in a 5% CO_2_ incubator. The cells were then removed, and CCK-8 reagent was added after 0 h, 24 h, 48 h, 72 h and 96 h. After incubation for 2 hours at 37°C, the OD value of each well was measured by an enzyme meter at 450 nm absorbance.

MCF-7-like organs with an average diameter greater than 50 μm were added to different gradients of tamoxifen at different concentrations and placed in a 37°C, 5% CO_2_ incubator. Cell Counting Lite 3D (Novozan) was then added and shaken vigorously for 5 minutes to allow sufficient lysis of the cell mass. The assay was performed after being left at room temperature for 25 minutes to stabilize the luminescence signal.

### Immunohistochemistry

The expression of RFC3 in primary and recurrent ER+ breast cancer tissues was studied by immunohistochemical analysis. Wax block sections were dewaxed using xylene before being subjected to hydrogen peroxide, citrate buffer, treated sections and heated for antigen repair. The primary antibody of the mouse monoclonal anti-RFC3 was then incubated and treated with phosphate buffer, and the secondary antibody was added. The sections were then retreated by dropwise addition of DAB chromogenic solution, stained using hematoxylin and rinsed with water after staining. The sections were then dehydrated in ethanol and xylene and sealed with neutral gum. Finally, the sections were observed with a microscope and photographed, and the average RFC3 expression intensity was calculated using ImageJ.

### Overexpression plasmid transfection and siRNA transfection and flow cytometric cell cycle, apoptosis

Cells were added to DMEM and mixed with Lipofectamine 2000 transfection agent to make Lipofectamine 2000. A plasmid dilution was then prepared by mixing the RFC3 overexpression plasmid with serum-free medium. Lipofectamine 2000 was then mixed with serum-free medium and incubated in an incubator for 48 h. siRNA transfection was carried out in the same way as overexpression, and the RFC3 overexpression plasmid was simply replaced with siRNA.

### Flow cell cycle, apoptosis and Transwell assay

To detect the cell cycle, the desired cells were collected and fixed, then the cells are stained using a staining working solution configured using a mixture of Propidium Iodide PI staining solution (20×): RNase A (50×) = 100:5:2 ratio. Cell cycle assays were then performed using flow cytometry assays, according to the manufacturer’s instructions. DNA content was analysed using software such as CellQuest. To detect apoptosis, the reagents were replaced with Annexin V-FITC and 5 μl 7-AAD.

Cell migration and invasion ability were examined using an inserted Transwell with 8 μm wells. Cells were added to fetal bovine serum medium. Twenty-four hours later, noninvasive cells were removed from the upper chamber with a soft cotton swab, and cells that had invaded through the membrane into the lower chamber of the Transwell were fixed, stained, photographed and counted.

### Statistical analysis

The data were statistically analyzed using the built-in statistical module of GraphPad Prism 8, and the results were repeated three times. Analysis of variance (ANOVA) was used to compare the results of multiple experimental groups, and Student’s *t* test was used to compare the results of two experimental groups. *P* < 0.05 was considered statistically significant.

### Availability of data and materials

The datasets used during the current study are available from the corresponding author on reasonable request.

## RESULTS

### Successful establishment and identification of tamoxifen-resistant MCF-7R strains and MCF-7-like organs

**Figure 1:**
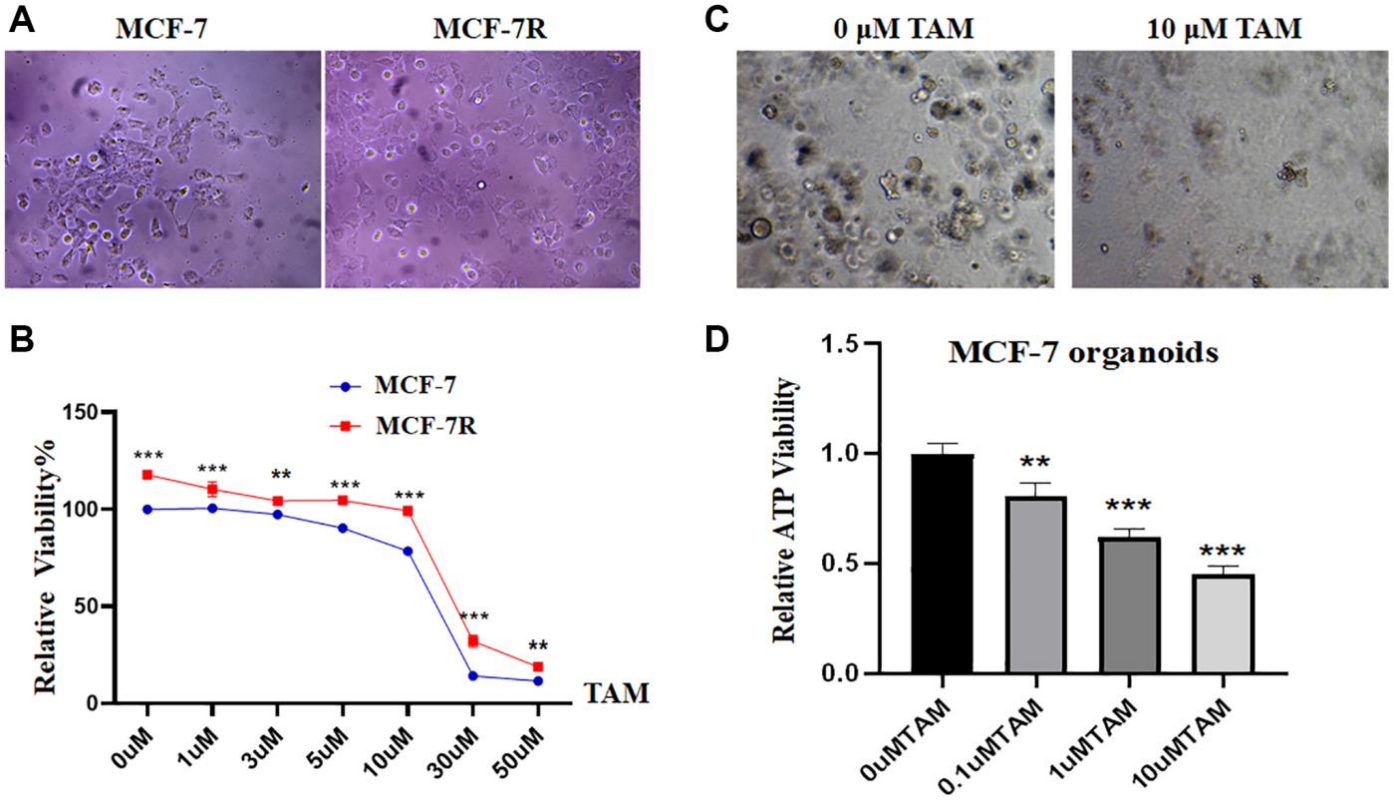
**Establishment and identification of tamoxifen-resistant cells and MCF-7-like organs.** (**A**) The morphology of the MCF-7R-resistant cell line is rounded and nearly spherical, whereas the MCF-7 parental cells are nearly spindle-shaped. (**B**) Proliferation rates of MCF-7 and MCF-7R cells after treatment with different concentration gradients of tamoxifen for 48 hours. (**C**) Growth of MCF-7-like organs in different concentrations of tamoxifen: intact growth at 0 μM, lysis and death at 10 μM. (**D**) ATP energy.

MCF-7R cells were successfully induced using a continuous induction technique with low concentrations of TAM. By inverted microscopy, we found that compared to the control group, the tamoxifen-resistant cell line had a more rounded, nearly spherical appearance, whereas the control group was spindle-shaped, showing a significant difference (Figure 1A). After CCK-8 experiments, the results showed that the cell proliferation rate of the resistant strain was significantly higher than that of the parental cell line under the same concentration of TAM treatment (Figure 1B, ^**^P < 0.01, ^***^P < 0.001), a result that provides important clues for further studies on TAM resistance in breast cancer. The results of the ATP activity assay showed that 10 μM tamoxifen inhibited the cell growth of MCF-7-like organoids, and the breast cancer-like organoids were lysed and killed (Figure 1C). The level of ATP energy expression in MCF-7-like organs decreased significantly with increasing tamoxifen concentration (Figure 1D, ^**^P < 0.01, ^***^P < 0.001).

### MCF-7-like organ transcriptome sequencing analysis and functional analysis results

**Figure 2:**
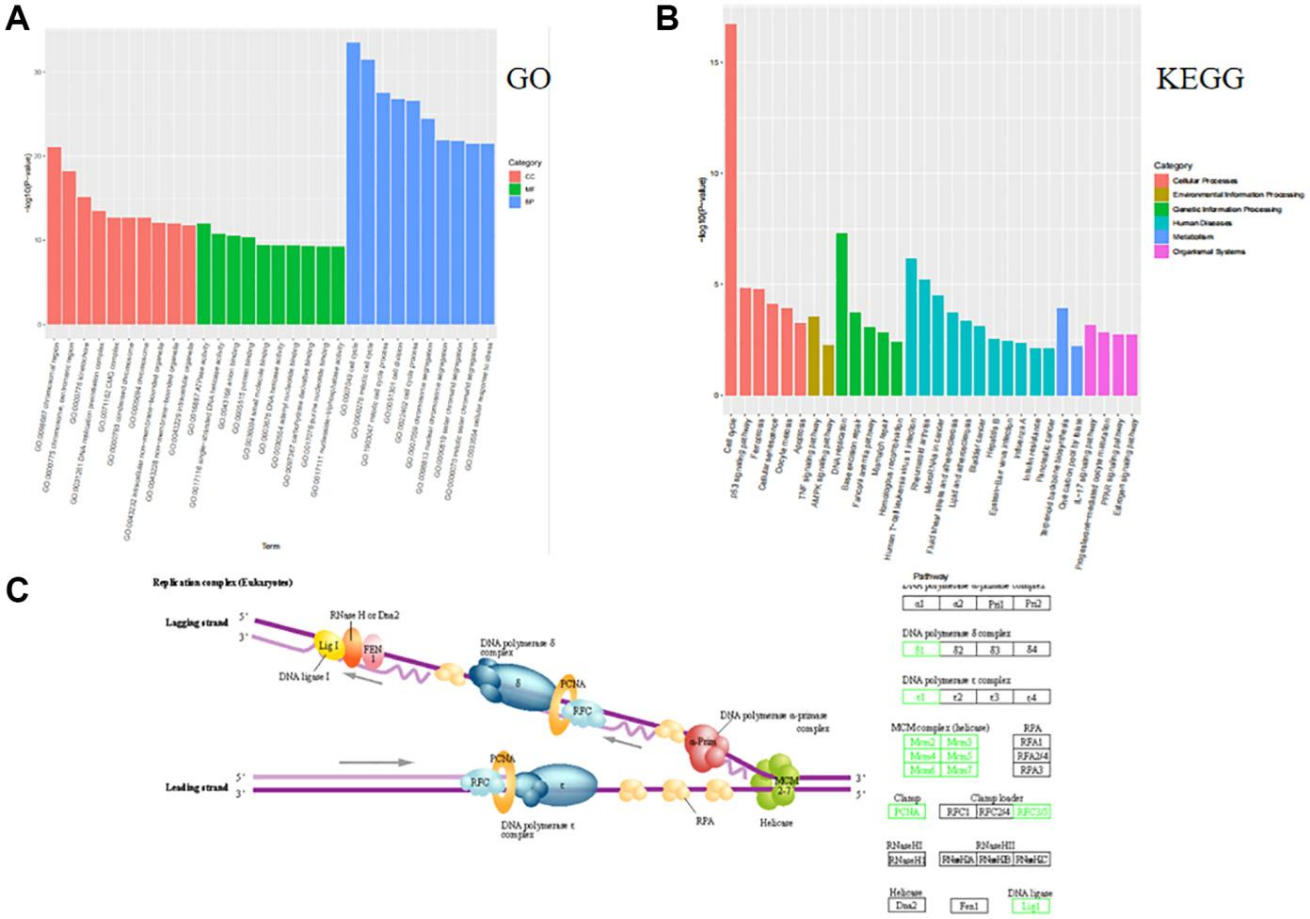
**MCF-7-like organ transcriptome sequencing analysis functional analysis of GO and KEGG.** (**A**) GO functional enrichment analyses of biological processes involved in breast cancer cellular process alterations in multiple ways, with the cell cycle approach in biological processes being the most significant. (**B**) The KEGG enrichment pathway is involved in multiple pathways in extracellular matrix-associated cellular process alterations, most notably through the cell cycle approach. (**C**) The RFC3, RFC5 and PCNA genes of the KEGG pathway are involved in the cell cycle through deoxyribonucleic acid replication.

The transcriptome sequencing analysis after tamoxifen treatment of MCF-7-like organs showed the highest number of cell cycle genes, and the GO bioprocess and KEGG cellular processes suggest that multiple signaling pathways in invasive breast cancer work together to regulate breast cancer progression, with the most significant involvement of the cell cycle approach (Figure 2A, 2B). The KEGG pathway map revealed that genes such as RFC3, RFC5 and PCNA are involved in the cell cycle pathway through deoxyribonucleic acid replication (Figure 2C).

### Increased MCF-7 cell cycle inhibition and γ-H2AX protein expression after TAM treatment

**Figure 3:**
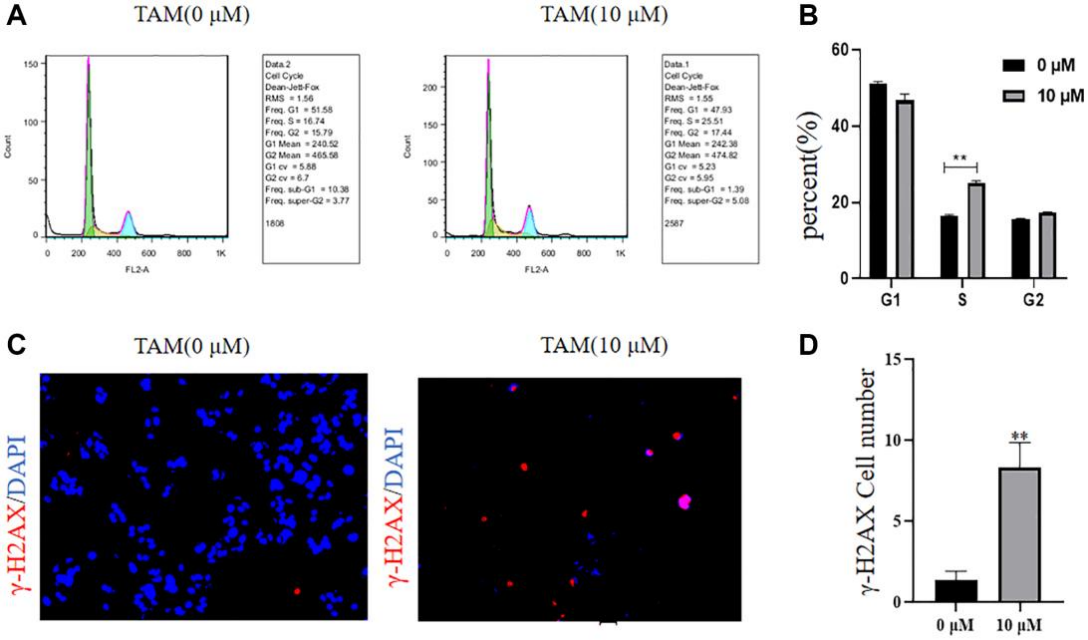
**Effects of different concentrations of tamoxifen on the MCF-7 cell cycle and γ-H2AX.** (**A**, **B**) The cell cycle of MCF-7 cells at a tamoxifen concentration of 10 μM was significantly increased by flow cytometric cell cycle assay compared to the proportion of S-phase of MCF-7 cells at a tamoxifen concentration of 0 μM. (**C**, **D**) IF detected that the γ-H2AX protein expression level of MCF-7 cells treated with 10 μM tamoxifen was significantly higher than that of MCF-7 cells treated with 0 μM tamoxifen.

We used a flow cycle assay to show that the S-phase of MCF-7 cells was increased and the cell cycle was significantly inhibited at 10 μM tamoxifen concentration compared to the control (Figure 3A, 3B, ^**^P < 0.01). Assays using IF showed that γ-H2AX protein expression levels were significantly increased in MCF-7 cells at a TAM concentration of 10 μM compared to those at 0 μM, indicating that TAM caused damage to the DNA of MCF-7 cells (Figure 3C, 3D, ^**^P < 0.01).

### Upregulation of RFC3 expression levels in MCF-7R and ER-positive breast cancer recurrent patient tissues

**Figure 4:**
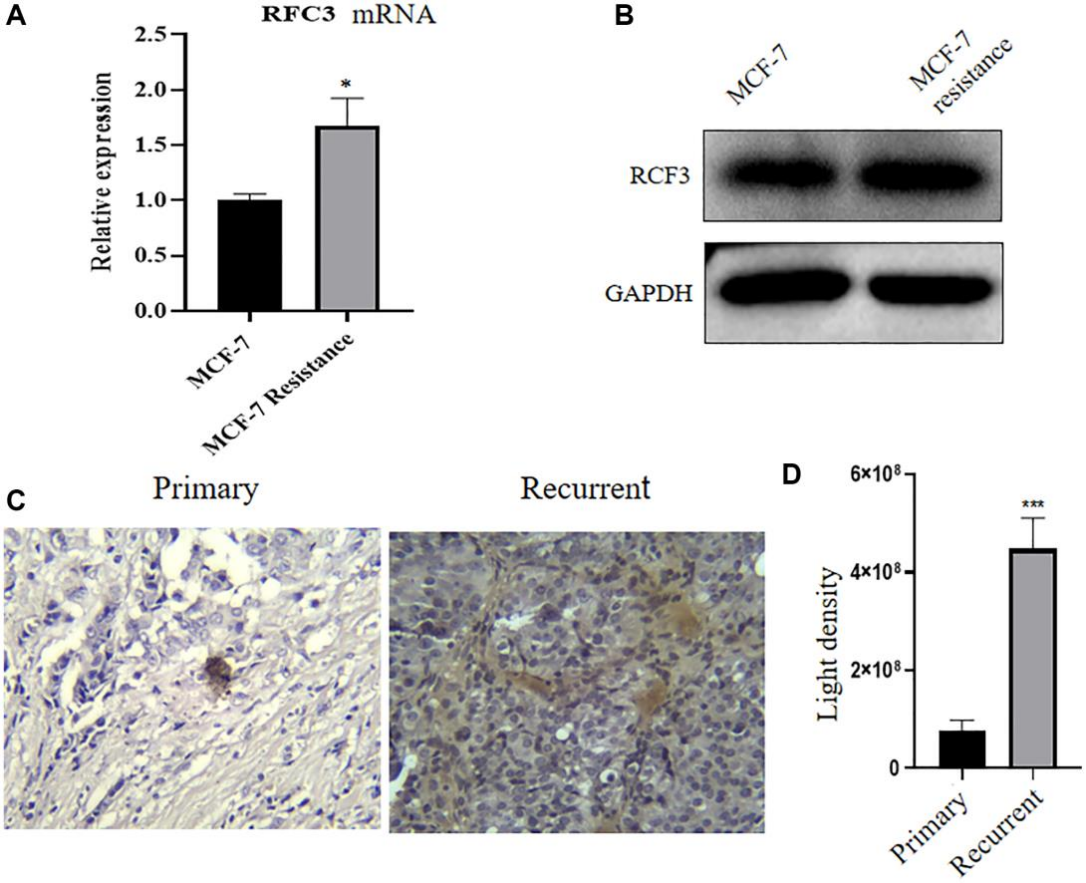
**RFC3 expression is upregulated in tamoxifen-resistant ER-positive breast cancer strains.** (**A**) qPCR results showed that RFC3 mRNA expression was upregulated in MCF-7R tamoxifen-resistant cells. (**B**) WB results showed that RFC3 protein was upregulated in MCF-7R tamoxifen-resistant cells. (**C**, **D**) IHC results showed that RFC3 protein expression was upregulated in pathological tissues of patients with relapse of endocrine therapy.

We examined the expression of RFC3 in TAM-resistant cell lines and tumor tissues. The expression of RFC3 mRNA and protein in cells by qPCR and western blotting and RFC3 protein expression in patient pathological tissues by IHC were detected, and the results showed that RFC3 mRNA expression was significantly upregulated in MCF-7R cells compared with MCF-7 cells (Figure 4A, ^*^P < 0.05). In MCF-7R cells, RFC3 protein expression levels were also significantly higher in MCF-7 cells than in MCF-7 cells (Figure 4B). In addition, RFC3 protein expression levels were also significantly increased in pathological specimens from patients with recurrent ER-positive breast cancer resistant to endocrine therapy (Figure 4C, 4D, ^***^P < 0.001).

### Elevated RFC3 expression in TAM-resistant cell microarrays

**Figure 5:**
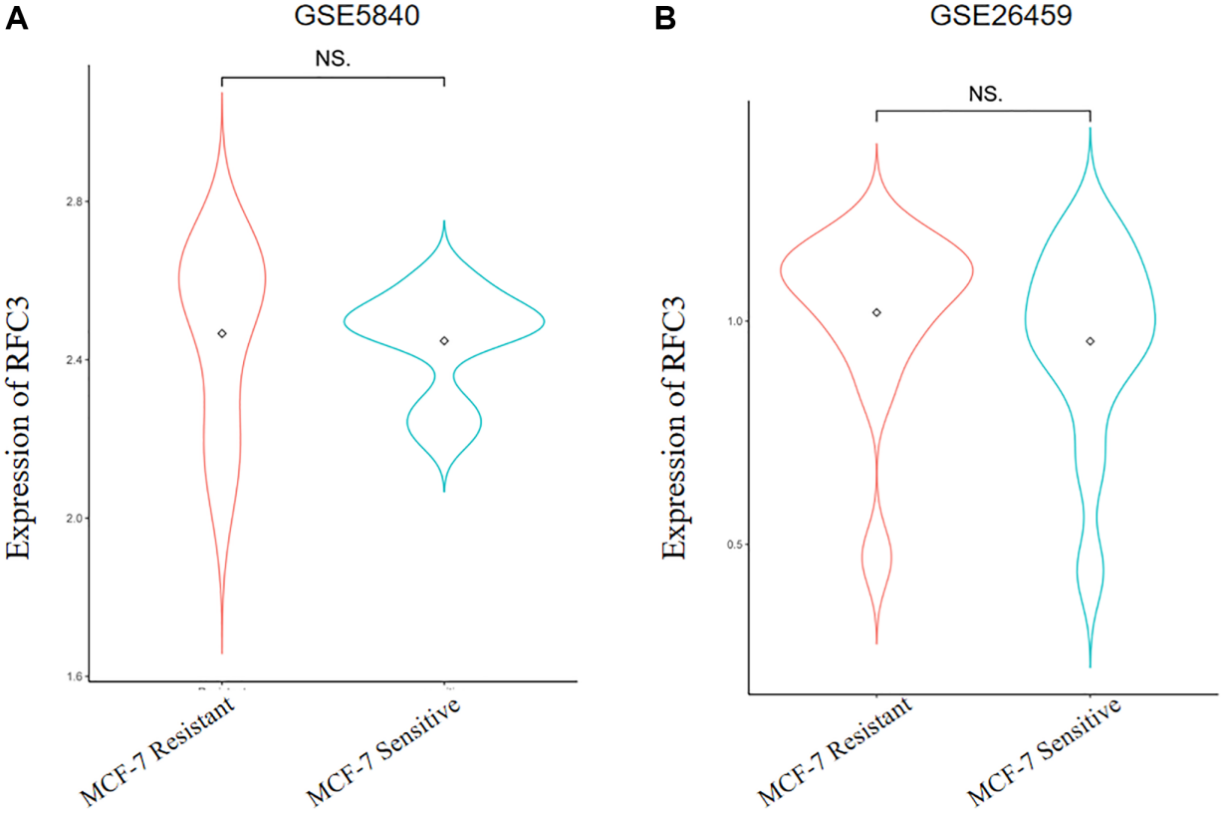
**Expression levels of RFC3 in tamoxifen-resistant strains of ER-positive breast cancer.** (**A**) Sequencing results show that RFC3 expression is upregulated in MCF-7R TAM-resistant cells. (**B**) Sequencing results showed that RFC3 expression was not significantly upregulated in MCF-7R tamoxifen-resistant cells.

We investigated the mechanism of TAM resistance in ER-positive breast cancer patients through the GEO database and obtained two different gene expression microarrays: GSE5840 and GSE26459. The differentially expressed genes were analyzed by R3.6.3 and its helper package. The results showed that RFC3 was upregulated in TAM-resistant MCF-7R cells (Figure 5A, 5B, *P* > 0.05).

### RFC3 expression affects the prognosis of patients with ER-positive breast cancer

**Figure 6:**
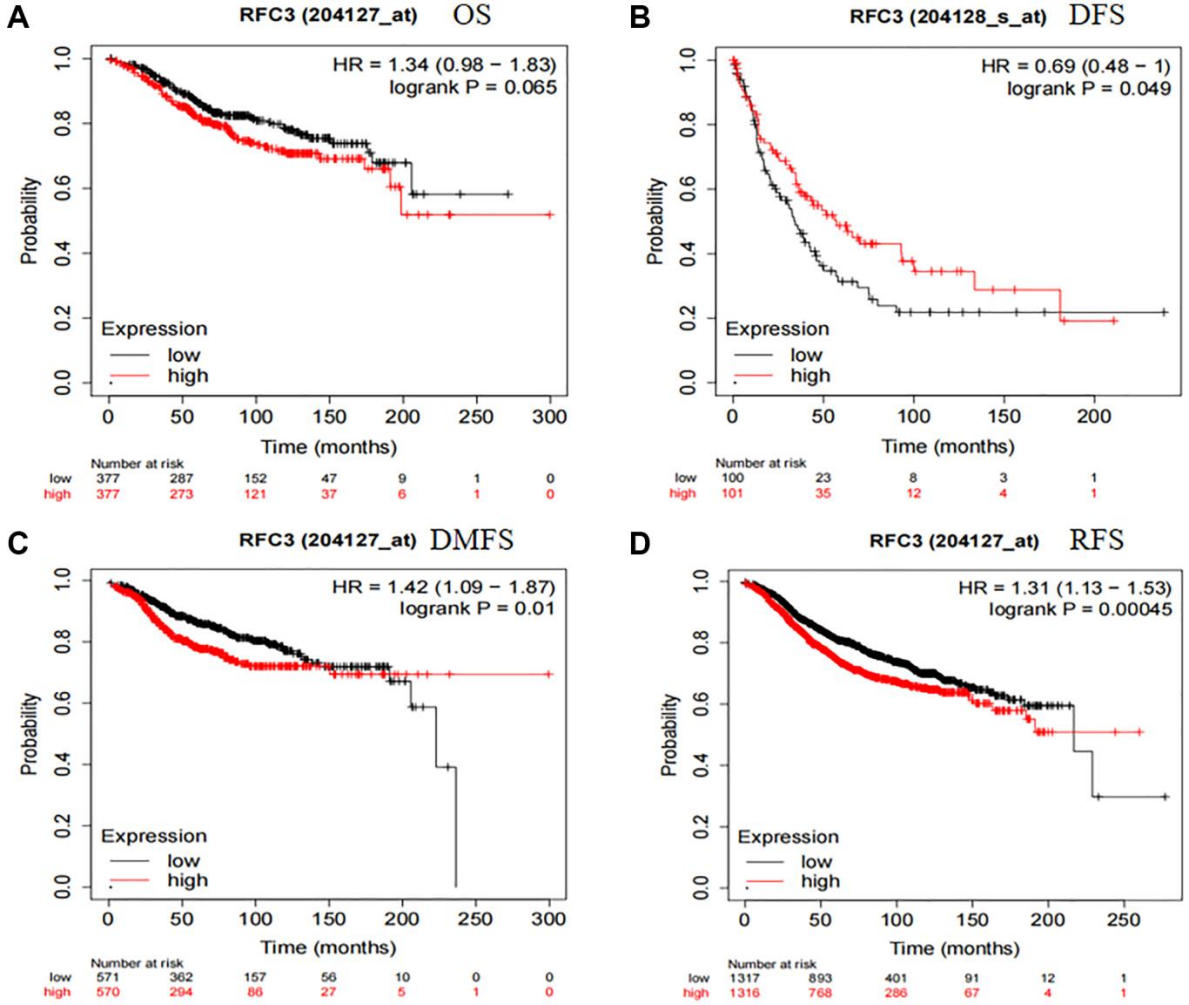
**RFC3 expression had an unfavorable effect on patients with ER-positive breast cancer.** (**A**) RFC3 expression had no effect on the OS of ER-positive patients. (**B**) Patients with high expression of RFC3 in ER-positive breast cancer had worse DFS. (**C**) Patients with high expression of RFC3 in ER-positive breast cancer had worse DMFS. (**D**) Patients with high expression of RFC3 in ER-positive breast cancer had worse RFS.

We then investigated the prognostic impact of RFC3 on patients with ER-positive breast cancer. The online Kaplan-Meier Plotter database (http://kmplot.com/analysis) was used to analyze the association between RFC3 expression levels and overall survival (OS), disease-free survival (DFS), disease-free survival (DFS), and distant metastasis-free survival (DMS) in ER-positive breast cancer patients), distant metastasis-free survival (DMFS) and relapse-free survival (RFS). The samples were divided into high and low expression groups based on the median cutoff value of RFC3 expression in ER-positive breast cancers. The results are shown in Figure 6. The level of RFC3 expression did not affect OS (HR 1.34, 95% CI 0.98–1.83, *P* = 0.065) and DFS (HR 0.69, 95% CI 0.48–1, *P* = 0.049) (Figure 6A, 6B). In contrast, patients with high RFC3 expression levels had worse DMFS (HR 1.42, 95% CI 1.09–1.87, *P* = 0.01) and RFS (HR 1.31, 95% CI 1.13–1.53, *P* = 0.00045) (Figure 6C, 6D).

### Effects of TAM on MCF-7 overexpressing RFC3

**Figure 7:**
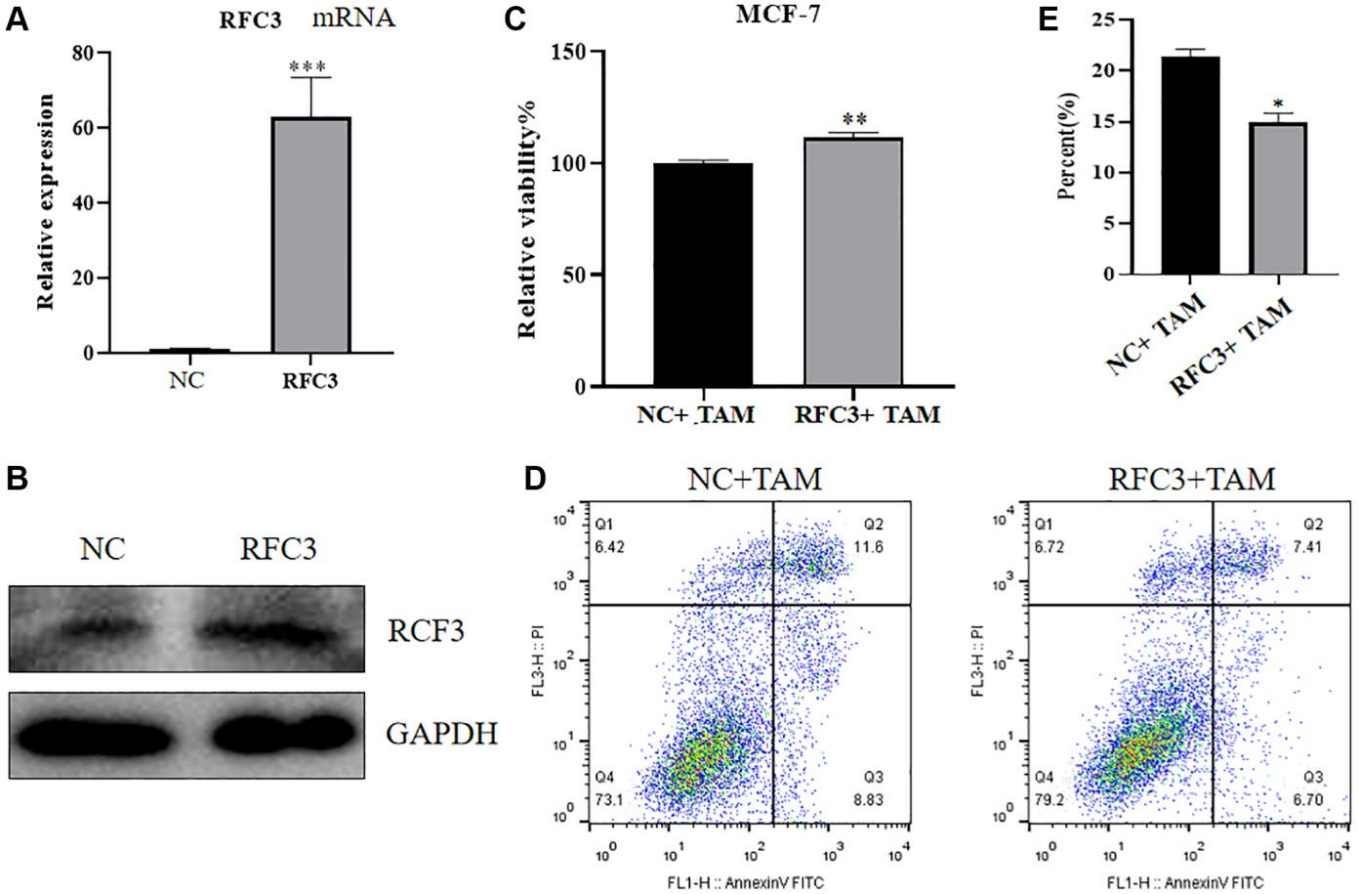
**Effect of RFC3 overexpression on MCF-7 cells.** (**A**) qPCR assay showed that RFC3 mRNA expression was significantly increased in MCF-7 cells overexpressing RFC3 compared to the control group. (**B**) WB assay showed that RFC3 protein expression was significantly increased in RFC3-overexpressing MCF-7 cells compared with the control group. (**C**) CCK-8 assay showed that the proliferation rate of RFC3-overexpressing MCF-7 cells was significantly increased after TAM treatment compared to the control group. (**D**, **E**) Flow cytometry apoptosis assay showed that apoptosis was significantly reduced in RFC3-overexpressing MCF-7 cells after TAM treatment compared to the control group.

To investigate the expression, proliferation and apoptosis of mRNA and protein in MCF-7 cells after upregulation of RFC3 levels in ER-positive breast cancer cells, we used qPCR and western blot assays to show that mRNA and protein expression levels were increased in RFC3-overexpressing MCF-7 cells after transfection of RFC3 overexpression plasmids compared to controls (Figure 7A, 7B, ^***^P < 0.001). Using the CC-K8 assay, it was found that the proliferation rate of RFC3-overexpressing MCF-7 cells was significantly increased compared to that of the control group, and the inhibitory effect of tamoxifen on the proliferation of RFC3-overexpressing MCF-7 cells was diminished compared to that of the control group (Figure 7C, ^**^P < 0.01). Using a flow cytometric assay, it was found that the proportion of apoptotic death in RFC3-overexpressing MCF-7 cells was less than that in the control group, indicating that the pro-apoptotic effect of tamoxifen on RFC3-overexpressing MCF-7 cells was diminished compared to the control group (Figure 7D, 7E, ^*^P < 0.05).

### Effects of TAM on knockdown of RFC3 in MCF-7R

**Figure 8:**
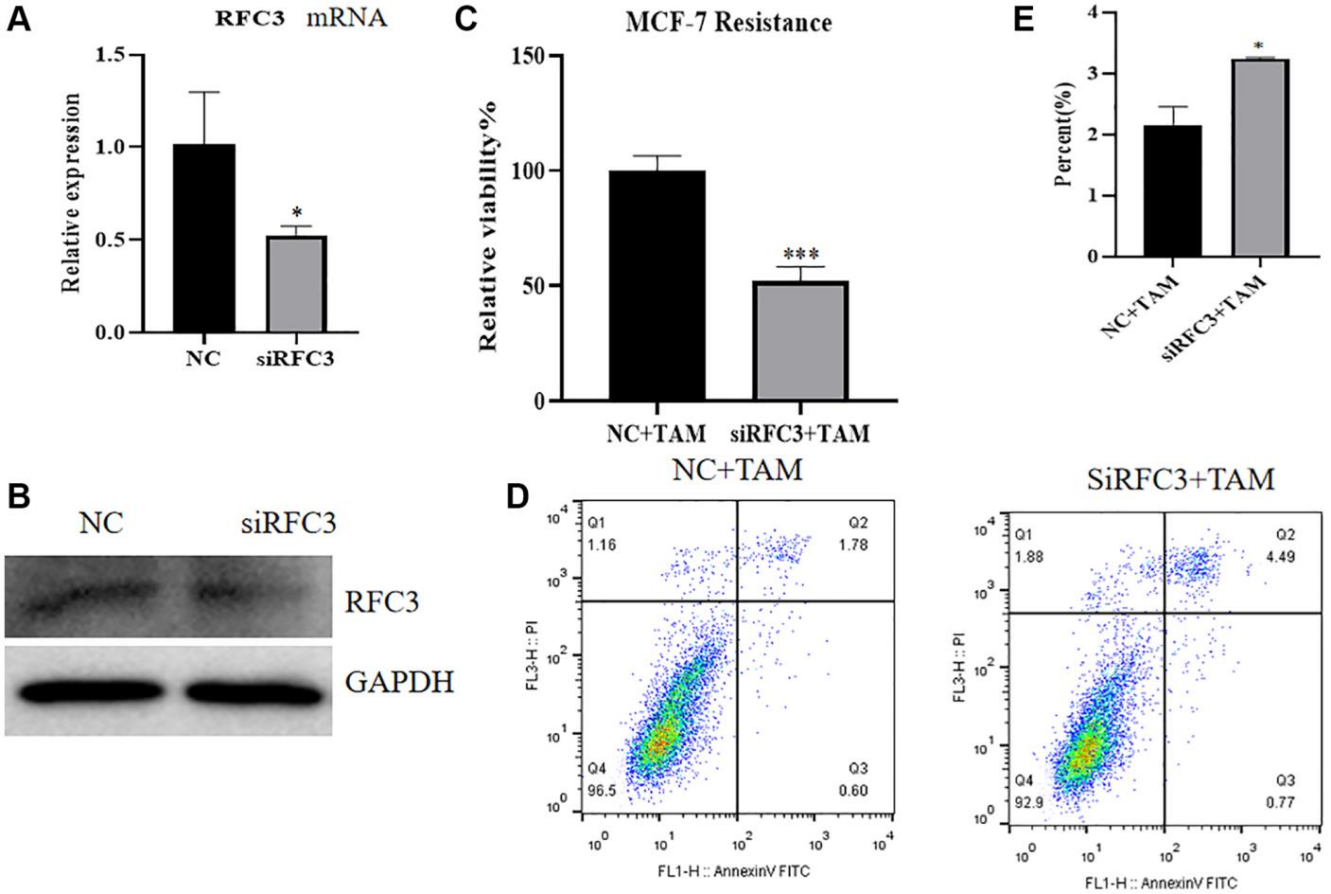
**Effect of RFC3 knockdown on MCF-7R cells.** (**A**) qPCR assay showed that RFC3 mRNA expression was significantly reduced in MCF-7R cells with knockdown of RFC3 compared to the control group. (**B**) WB assay showed that RFC3 protein expression was significantly reduced in MCF-7R cells with RFC3 knockdown compared to control cells. (**C**) CCK-8 assay showed that the proliferation rate of RFC3-knockdown MCF-7R cells treated with TAM was significantly reduced compared with the control group. (**D**, **E**) Flow cytometry apoptosis assay showed that apoptosis was significantly increased in MCF-7R cells with RFC3 knockdown after TAM treatment compared to the control group.

To investigate the expression, proliferation and apoptosis of mRNA and protein in MCF-7R cells after knockdown of RFC3 levels in ER-positive breast cancer resistant cells, QPCR and western blot assays showed that compared with drug-resistant MCF-7R cells, siRNA transfection of MCF-7R drug-resistant cells resulted in a reduction in RFC3 mRNA and protein expression levels (Figure 8A, 8B, ^*^P < 0.05). The CCK-8 assay showed that siRNA transfection of MCF-7R-resistant cells showed a significant decrease in proliferation rate compared to the control group, and siRNA transfection of MCF-7R-resistant cells resulted in enhanced proliferation inhibition of tamoxifen on MCF-7R-resistant cells compared to the control group (Figure 8C, ^***^P < 0.001). Flow cytometric assays showed that the proportion of apoptosis was increased in siRNA-transfected MCF-7R-resistant cells compared to control cells, and the proapoptotic effect of tamoxifen on MCF-7R-resistant cells after RFC3 knockdown was enhanced in siRNA-transfected MCF-7R-resistant cells compared to control cells (Figure 8D, 8E, ^*^P < 0.05).

### Cell cycle effects of TAM on RFC3 overexpressing MCF-7 cells and RFC3 knockdown MCF-7R

**Figure 9:**
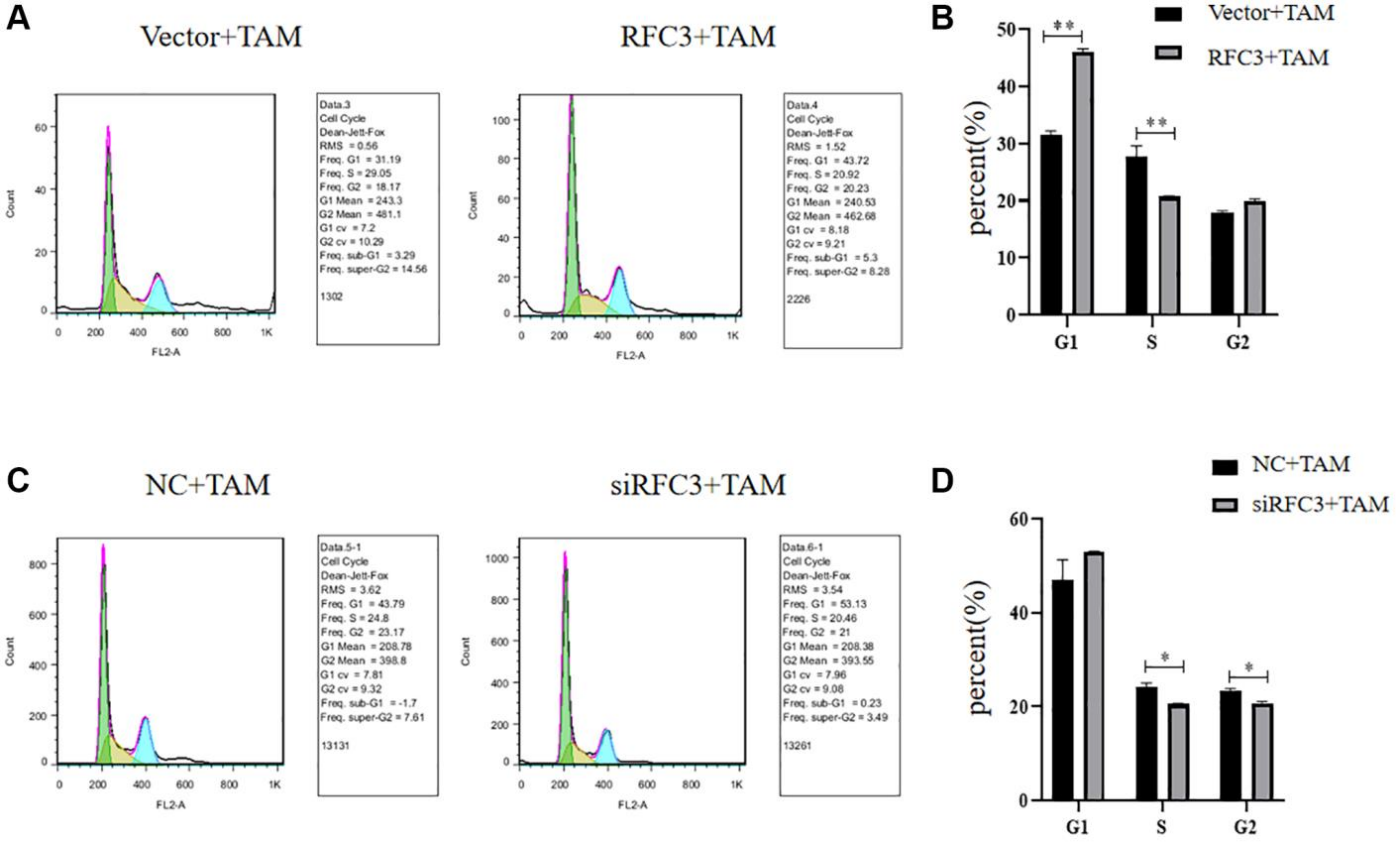
**Cell cycle of RFC3-overexpressing MCF-7 cells and MCF-7R cells with RFC3 knockdown.** (**A**, **B**) Flow cytometry experiments showed that the proportion of MCF-7 cells with RFC3 overexpression decreased in S-phase and increased in G1-phase, and cell cycle inhibition was reduced in TAM-treated MCF-7R cells compared with the control group. (**C**, **D**) Flow cytometry experiments showed that the proportion of MCF-7R cells with knockdown of RFC3 decreased in S-phase, decreased in G2-phase, and enhanced cell cycle inhibition after TAM treatment compared with the control group.

To investigate whether the cell cycle inhibition of ER-positive breast cancer cells by TAM is affected by the level of RFC3 expression, we showed by flow cytometric cell cycle assay that, compared with the control group, the proportion of MCF-7 cells in S-phase decreased and the proportion of MCF-7 cells in G1-phase increased after RFC3 overexpression in MCF-7 cells treated with TAM. The proportion of S-phase and G1-phase was decreased and cell cycle inhibition was reduced in RFC3 overexpressed MCF-7 cells compared to control cells (Figure 9A, 9B, ^**^P < 0.01), whereas the proportion of S-phase and G2-phase was decreased and cell cycle inhibition was enhanced in RFC3 knockdown treated MCF-7R resistant cells by TAM compared to control cells by siRNA transfection (Figure 9C, 9D, ^*^P < 0.05).

### RFC3 affects the migration of TAM-resistant cells in ER-positive breast cancer

**Figure 10:**
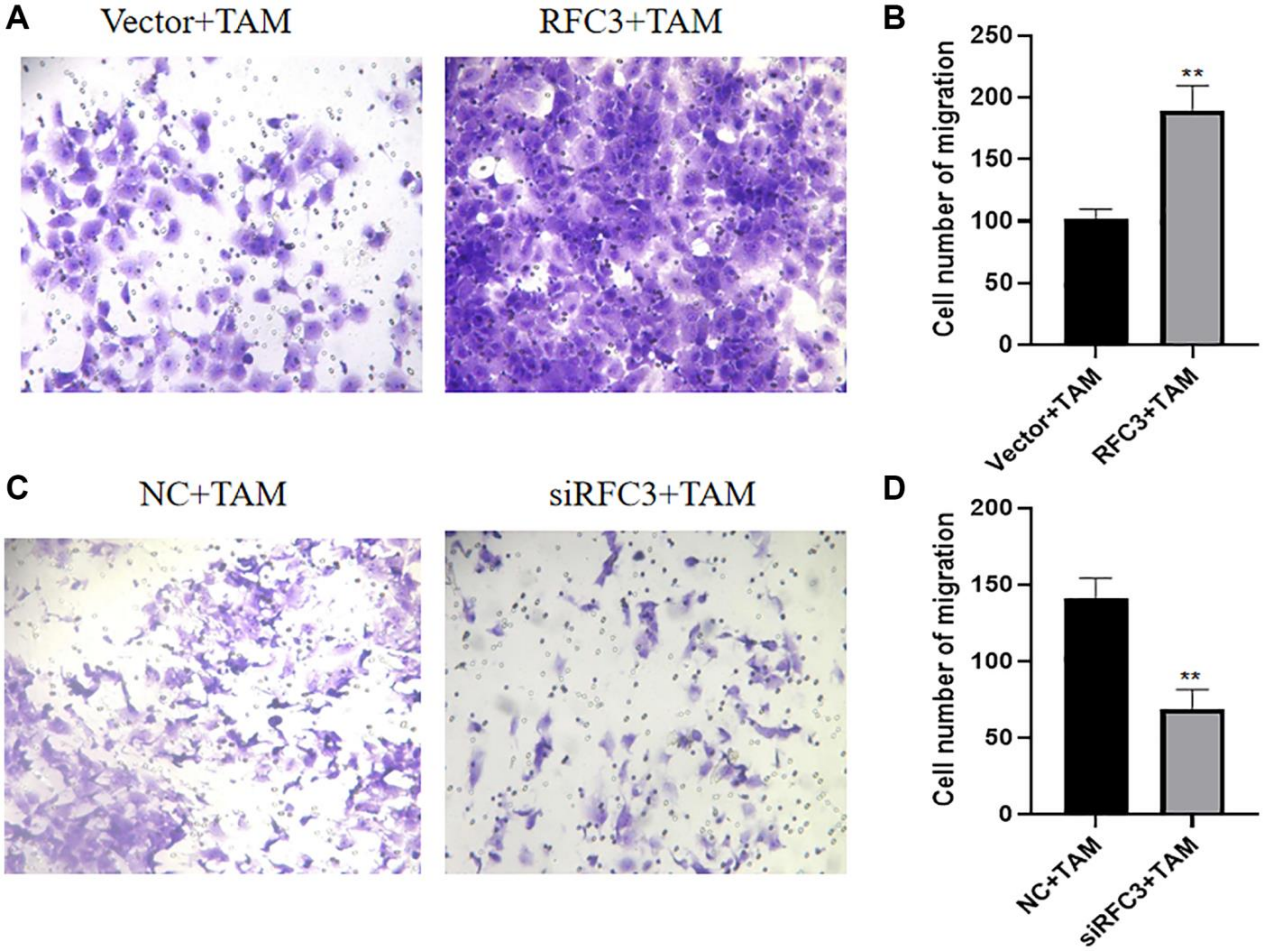
**RFC3 affects the migration of TAM-resistant cells in ER-positive breast cancer.** (**A**, **B**) Transwell assay showed a significant increase in the number of putative cells in the MCF-7 group with RFC3 overexpression compared to the control group. (**C**, **D**) Transwell experiments showed that the number of effaced cells was significantly reduced in the MCF-7R group with RFC3 knockdown compared to the control group.

Metastasis of tumors is one of the markers of tamoxifen treatment resistance. Therefore, we further investigated the effect of RFC3 expression level on the migratory ability of TAM-resistant cells. By Transwell migration assay, we examined the changes of cell migration ability before and after RFC3 overexpression in MCF-7 and before and after RFC3 knockdown in MCF-7R, respectively. The results showed that the number of cells penetrating the bottom of Transwell was significantly increased in MCF-7 with RFC3 overexpression compared to the control group (Figure 10A, 10B, ^**^P < 0.01). The number of cells penetrating the bottom of Transwell was significantly decreased in the MCF7-R group with RFC3 knockdown compared to the control group (Figure 10C, 10D, ^**^P < 0.01). It indicates that the expression of RFC3 in ER-positive breast cancer TAM-resistant cells affects the migration ability of breast cancer cells, and the migration ability of breast cancer cells was enhanced when RFC3 was overexpressed, while the migration ability of breast cancer resistant cells was reduced after RFC3 was knocked down.

## DISCUSSION

As a result of the present study, we established an MCF-7R-resistant cell line, and the induced MCF-7R cells showed a rounded shape, close to a spherical shape, while their parental cells were closer to a spindle shape. According to previous studies, MCF-7R morphology undergoes spherical changes when ERα is knocked down [27]. Previous studies have reported the use of CCK-8 to detect the proliferation of pancreatic cancer cells [28]. This was confirmed by the results of the current experiment, where CCK-8 showed that the cells proliferated at a higher rate after TAM induction than before induction. Organoids have previously been reported in the literature to be generated from stem cells *in vitro* through a three-dimensional culture environment [29]. We obtained MCF-7 organoids *in vitro* in culture using breast cancer organoid medium and cell passaging techniques. Since the successful introduction of intestinal organoids in 2009, tremendous and long-lasting advances have been made in organoid culture technology, which is gradually becoming the most innovative and cutting-edge technology in the biomedical field [30]. Previous studies have shown that RFC3 not only promotes DNA replication, repair, and cell proliferation but also regulates cell cycle checkpoints. RFC3 plays an important role in all stages of eukaryotic cells [31]. The DNA damage repair protein H2AX can play a role in regulating DNA repair through homologous recombination. h2AX contributes to DNA repair in mammals. In mammals, H2AX contributes to DNA repair and regeneration [32]. We analyzed the transcriptome of MCF-7-like organs by sequencing bioenrichment analysis and concluded that there are more cell cycle genes and that genes such as RFC3, RFC5 and PCNA are involved in the cell cycle. Flow cytometric assays showed that the S-phase of MCF-7 cells was blocked by tamoxifen treatment, and IF assays showed that the cell cycle repair protein γ-H2AX was increased by tamoxifen treatment. These results confirm the involvement of RFC3 in the cell cycle through DNA replication. The expression of RFC3 was significantly higher in tamoxifen-resistant cell lines than in sensitive lines, and the expression level of RFC3 was significantly increased in recurrent breast cancer tissues. RFC3 expression levels were significantly increased in the GEO database of breast cancer drug-resistant microarrays, suggesting that RFC3 may be involved in tamoxifen resistance. However, there was no statistically significant difference in the increased expression levels of RFC3 in the GEO database of breast cancer resistance microarrays, which may be related to the small sample size in the database and the small number of gene chips. Using the Kaplan-Meier Plotter database study, we analyzed the relationship between RFC3 expression levels and OS, DFS, DMFS and RFS in ER-positive breast cancer patients. The results showed that RFC3 expression levels had no effect on OS and DFS, but patients with high RFC3 expression levels had shorter DMFS and RFS, while DMFS and RFS were strongly associated with recurrence and distant metastasis in patients, and treatment recurrence or distant metastasis were the two main clinical manifestations of clinical resistance [33, 34].

The expression of RFC3 was then knocked down in MCF-7R cells, and the expression level of RFC3 was significantly reduced compared to that in the control group. The proliferation inhibitory effect of tamoxifen on MCF-7R cells with RFC3 knockdown was enhanced, and the proapoptotic ability of tamoxifen on MCF-7R cells with RFC3 knockdown was improved. Mesenchymal markers and epithelial markers in TNBC cells are significantly reduced when RFC3 levels are reduced, thereby significantly attenuating cell proliferation. Migration and invasion and overexpression of RFC3 in TNBC show metastasis, progression and poor prognosis [35]. Overexpression of RFC3 in the presence of low RFC3 abundance in MCF-7 cells by CCK-8 assay and flow cytometry showed that overexpression of RFC3 reversed the inhibition of proliferation of tamoxifen in MCF-7 cells after overexpression treatment. The above results complement each other in the phenotypic changes in MCF7R induced by RFC3 knockdown, indicating that RFC3 is not only associated with proliferation and apoptosis in ER-positive breast cancers but also strongly linked to tamoxifen resistance in ER-positive breast cancers. It was demonstrated that RFC3 overexpression could effectively reduce the inhibitory ability of tamoxifen against ER-positive breast cancer, while RFC3 knockdown could effectively enhance the inhibitory ability of tamoxifen against drug-resistant ER-positive breast cancer. The flow cytometric assay showed that overexpression of RFC3 attenuated the cell cycle inhibition of tamoxifen against MCF-7 after overexpression treatment, and knockdown of RFC3 enhanced the cell cycle inhibition of tamoxifen against MCF-7 after overexpression treatment. This demonstrates that RFC3 is associated with the cell cycle and can influence endocrine resistance in breast cancer by affecting the cell cycle. This is consistent with previous studies in which downregulation of RFC3 in ovarian cancer OVCAR-3 cells may lead to S-phase arrest [21]. Metastasis of cancer is a serious challenge because it leads to invasion and spread of tumor cells, which can metastasize from the primary site to the whole body and may lead to severe organ failure, resulting in patient death [36]. In the present study, we used Transwell migration assays to assess the spreading and invasive ability of breast cancer cells and found that RFC3 overexpression enhanced the spreading and invasive ability of breast cancer cells. The results showed that RFC3 overexpression enhanced the spread and invasive ability of breast cancer cells, while RFC3 knockdown diminished the spread and invasive ability of resistant breast cancer cells, which fully demonstrates that the expression level of RFC3 affects the spread and invasive ability of breast cancer cells.

In conclusion, our study shows that RFC3 upregulation, positively correlates with overall disease-free and metastasis-free survival. Furthermore, knockdown of RFC3 expression severely impairs the proliferation, invasion and cell cycle progression of drug-resistant cells in breast cancer. This study suggests that RFC3 may play a key role in tumor metastasis and could be a novel diagnostic marker and potential therapeutic target for drug resistance in breast cancer. However, we acknowledge that more clinical samples and animal experiments are needed to verify the reliability of these results. In addition, more work needs to be done in the future to find out the exact regulatory network of RFC3 and novel inhibitors for RFC3.

## CONCLUSION

Taken together, our findings suggest that RFC3 may be involved in endocrine resistance in breast cancer through DNA replication in the cell cycle and is significantly associated with metastasis and progression of breast cancer.
